# ETHIOPIAN CENTENARIANS: HEALTH CONDITIONS AND SENSORY/COGNITIVE FUNCTIONALITY

**DOI:** 10.1093/geroni/igac059.2132

**Published:** 2022-12-20

**Authors:** Samson Chane, Margaret Adamek

**Affiliations:** Bahir Dar University, Bahir Dar, Amhara, Ethiopia; Indiana University, Indianapolis, Indiana, United States

## Abstract

Globally, the centenarian population is expected to increase in the coming three decades reaching 3 million by 2050. In Ethiopia, like other Sub-Saharan African countries, information about the health condition, sensory and cognitive functionality of centenarians is scanty. This study examined the health conditions and sensory/cognitive functionality of Ethiopian centenarians. A qualitative case study design was employed. Nine centenarians (1 woman, 8 men) between age 100 and 108 were identified using snowball sampling. Data were generated through in-depth interviews and analyzed using descriptive analysis. All 9 were Orthodox Christians and lived in rural areas either with their wives (3) or other relatives. Four centenarians could read and write, two attended grade eight and nine, and three were non-literate. Before retirement the centenarians engaged in different occupations as farmers (4), shoemaker, government employee, school teacher, priest, “bounty hunter” and mechanic. Most perceived that their sensory and cognitive functionality was normal until their later 90s except for minor complaints. Centenarians faced walking difficulty (5), joint tightness (4), back pain (4), fatigue, dental issues, stuttering, hypertension (3), insomnia, diabetes, anorexia, severe headache, constipation and anxiety. Two centenarians identified themselves as escapers. Centenarians faced hearing defect (4), sight problems, and a decrease in taste and smell. Two reported proper functioning of their sensory organs. Despite various health problems, the Ethiopian centenarians aspired to live longer resiliently. Findings call for further study and the need for social workers, caregivers, and health care practitioners to consider the health conditions, sensory and cognitive functionality of centenarians.

