# Correction to: ETHIOPIAN CENTENARIANS: HEALTH CONDITIONS AND SENSORY/COGNITIVE FUNCTIONALITY

**DOI:** 10.1093/geroni/igad025

**Published:** 2023-03-27

This is a correction to: Samson Chane, Margaret Adamek, ETHIOPIAN CENTENARIANS: HEALTH CONDITIONS AND SENSORY/COGNITIVE FUNCTIONALITY, Innovation in Aging, Volume 6, Issue Supplement_1, November 2022, Pages 565–566, https://doi.org/10.1093/geroni/igac059.2132

In the originally published version of this abstract, affiliation 1 was erroneous and it has now been corrected to Bahir Dar University, Bahir Dar, Amhara, Ethiopia.

